# Reactions to policy action: socio-political conditions of backlash to climate change policy

**DOI:** 10.1007/s11077-025-09578-5

**Published:** 2025-05-29

**Authors:** James Patterson, Ksenia Anisimova, Jasmin Logg-Scarvell, Cille Kaiser

**Affiliations:** Copernicus Institute of Sustainable Development, Utrecht University, Utrecht, The Netherlands

**Keywords:** Policy backlash, Political backlash, Policy conflict, Policy design, Policy implementation

## Abstract

Public policymaking on issues requiring ambitious yet socially and economically costly action can face backlash from target groups and wider audiences, threatening policy adoption and durability. As an abrupt negative reaction to policy action, backlash is challenging to study and requires distinctive analytical approaches. This is especially pressing for climate change mitigation policy, which faces growing yet dispersed empirical experiences of backlash. We develop a framework to study the socio-political conditions (economic, cultural, practical) under which backlash to climate policy occurs to enable comparative empirical analysis. We posit that backlash arises from significant incongruence between policy action and its socio-political context across one or more of these dimensions. We illustrate this approach using three cases of backlash to carbon pricing policy in Canada, France, and Mexico, revealing different ways in which incongruence can arise. Our analysis highlights the need for configurational explanations and a policy-in-context perspective when studying contentious reactions to policy action.

## Introduction

Public policymaking on issues requiring ambitious yet socially and economically costly action, such as climate change, risk backlash from target groups and wider audiences threatening policy adoption and durability. ‘Hard’ or coercive^1^ policies such as regulation, taxes, and phase-outs – i.e., those involving “the threat of sanction or the use of force” (Mansbridge, 2012, p.4) or “negative incentives” (Schulze, 2021, p.10) – may be needed but can be deeply contested and vulnerable to backlash. Policy backlash involves “an abrupt and forceful negative reaction by a significant number of actors within a political community seeking to reverse a political development” (Patterson, 2023, p.69). This can derail policy action, undermine broader policy agendas, and render issues toxic in future rounds of debate (Patashnik, 2023), potentially also undermining public confidence in the capacity of the state to address difficult long-term problems. Understanding how and why backlash occurs is, therefore, crucial for policy science and practice.

Backlash risk is observed in many domains (Patashnik, 2023) including climate change (Patterson, 2023), migration (Claassen & McLaren, 2022), gender equality (Mansbridge & Shames, 2008), and civil rights (Encarnación, 2020). However, the conditions under which backlash occurs remain unclear. Backlash reactions may stem from perceptions of costs (Mildenberger et al., 2022; Patashnik, 2023, p.10), policy meanings forged in political debates (Maor & Capelos, 2023; Stone, 2012), perceived impacts on everyday life (Bulkeley et al., 2016; Weaver, 2014), or perceived policy overreach (Jones et al., 2019; Patashnik, 2019). Hence, both costs and norms of policy action may matter. Backlash might be triggered by seemingly minor policy changes, such as incremental increases in fuel taxes (Driscoll, 2023) or public transport fares (Palacios-Valladares, 2020). Yet, both incremental and structural policy shifts may fail to trigger backlash, even when it might be notionally expected such as for major new climate policy under the United States’ Inflation Reduction Act of 2022 (Meyer, 2022; Patashnik, 2023). Backlash likely depends on the interplay between policy action and its socio-political context, but under which conditions it occurs remains unclear.

Backlash is under-studied in policy sciences yet connects with existing lines of thinking. A policy design perspective may view backlash as an acute implementation challenge. However, this risks narrowing its focus to aspects such as design features, administration, or political will (Tosun & Schaub, 2023) and implies that it is a problem to solve rather than a dependent variable to explain. A policy process perspective, such as the Advocacy Coalition Framework and Punctuated Equilibrium Theory, may view backlash as a type of political struggle between rival policy proponents (Weible & Sabatier, 2017). Although, this perspective typically explains policy *change* (where policy is the unit of analysis), rather than explaining *reactions* to policy action (where reactions of target groups and wider audiences to policy action is the unit of analysis).^2^ Alternatively, a policy feedback perspective may view backlash as a form of rapid negative feedback within a wider political arena (Patashnik, 2019, 2023). From another angle, a policy conflict perspective (Kagan et al., 2023; Weible & Heikkila, 2017) highlights questions of how and why contention occurs, offering a view of backlash as an intense, reactive, and abrupt form of policy conflict. Ways of analyzing the socio-political conditions under which backlash arises in specific policy domains are needed.

We therefore ask: Under which socio-political conditions does backlash to climate policy arise? We aim to enable comparative empirical analysis to explain growing (although sporadic) cases of backlash in the climate change policy domain (Anisimova & Patterson, 2024). We develop a framework of socio-political conditions spanning economic, cultural, and practical dimensions, positing that backlash arises from significant incongruence between policy action and its socio-political context across one or more of these dimensions. Incongruence refers to a significant perceived mismatch between the demands of policy action and target groups’ capacity to accommodate these demands. We illustrate our approach using three cases of backlash to climate policy in Canada, France, and Mexico. Variations in how incongruence arises in these cases suggests the need for configurational explanations of backlash. We thereby contribute to studying backlash as an open-ended and contextually embedded reaction (Patterson, 2023, p.68), reflecting a need to expand policy-centered analysis towards a policy-in-context perspective when studying contentious reactions to policy action. This could also broadly inform the development of analogous frameworks in other domains.

## Locating backlash in policy sciences

First, we locate policy backlash as a *reaction* to policy action in relation to policy sciences. Reflecting on empirical patterns of backlash to climate policy then further helps to show how it manifests empirically, informing the development of our analytical approach.

### Reaction to policy action

**Fig. 1:**
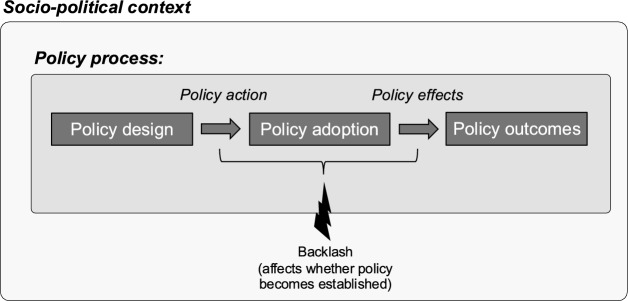
Position of backlash in the policy process

Backlash is an abrupt negative reaction that occurs in response to a policy action during or following policy adoption (Fig. 1), threatening policy establishment prior to longer-term questions of entrenchment (Hacker & Pierson, 2019). Backlash thereby intervenes between policy design and outcome phases of the policy process, calling into question whether a policy even proceeds. Importantly, backlash requires moving beyond an implementation focus (i.e., how to implement a designed policy) to a reaction-focused perspective (i.e., understanding why negative reactions occur). For example, whether target groups and wider audiences accept costs depends not only on individual assessments of utility but also on socially constructed perceptions and meanings generated within complex contexts (following Stone, 2012). Reactions may be driven by diverse and contingent factors that shape how policy is perceived. Complex causation might also be important. While consistent conditions that increase backlash risk can be identified (e.g., Patashnik, 2023), we underscore the need for open-ended attention to socio-political context in doing so.

Negative reactions to policy action are rarely foregrounded but resonate across policy theories. For example, the Advocacy Coalition Framework (ACF) and Punctuated Equilibrium Theory (PET) highlight political struggles among rival policy proponents during and after policy change, driven by beliefs (ACF) or policy images (PET) (Weible & Sabatier, 2017). They typically focus on explaining policy *change* (where policy is the unit of analysis) rather than *reactions* to policy action (where reactions by target groups and wider audiences to a policy action is the unit of analysis). Nonetheless, Jones et al., (2019) combine PET with policy feedback in observing “conservative reaction” to policy expansion in the United States involving multiple forms of discontent (e.g., anti-tax sentiments, resentment, clash of values). From another angle, the Multiple Streams Framework highlights how “problematic policy preferences … are not fixed and exogenously given but emerge during (inter)action” (Herweg et al., 2017, p.19), and that “value acceptability” and “public acquiescence” (among others) impact policy survival (Herweg et al., 2017, p.23–24). More broadly, interpretive policy scholars emphasize how perceptions and symbolic meanings of policy shape responses to it (Stone, 2012).

Another key approach is policy feedback, which highlights how feedback instigated by a policy can affect ongoing struggles at the post-adoption stage by reshaping institutions and preferences (Jordan & Moore, 2020; Lockwood, 2022; Patashnik & Zelizer, 2013). For example, feedback can undermine a policy by eroding support or changing incentives (Jacobs & Weaver, 2015) or reshaping advocacy coalitions (Schmid et al., 2020). Oberlander and Weaver (2015, p.41) suggest that undermining (negative) socio-political feedback stems from perceived concentrated losses, associated grievances, and shifts in coalitions and policy agendas. More broadly, Patashnik and Zelizer (2013, p.1072) emphasize the role of “an uncertain and contentious political environment” in conditioning policy feedback.

Building on these ideas, Patashnik (2023) develops a comprehensive view of policy backlash as countermobilization whereby “policies construct, fortify or mobilize opposition forces rather than build supportive coalitions” (Patashnik, 2023, p.171). While policy feedback is often seen as gradual, Patashnik posits backlash as rapid negative feedback involving countermobilization by mass publics and/or elites (e.g., politicians, businesses, labor unions) against perceived policy impacts (also see: Patashnik, 2019). Patashnik (2023) argues that backlash arises due to the combination of policy attributes (concentrated or general costs, threats to incumbent actors, negative judgments about others who stand to gain, and/or policy overreach), target groups’ capacity to mobilize, and political opportunities (especially division among elites, and availability of potential allies). This brings together policy design and contextual factors and acknowledges contingency (Patashnik, 2023, p.20–21). Yet, the role of context could perhaps be even wider in certain policy domains such as climate change. In particular, additional domain-specific aspects may need to be considered when comparing diverse cases or when unpacking why certain perceptions that come to underpin backlash arise. Elaborating on these aspects can help to enrich our understanding of why episodes of backlash erupt in specific domains.

Finally, a policy conflict frame is useful because it questions how and under which conditions policy contestation occurs (Kagan et al., 2023; Weible & Heikkila, 2017). Through this lens, policy backlash is an intense form of policy conflict that occurs as a reaction against a policy that has been (or imminently will be) adopted,^3^ seeking to remove or reverse it. Weible and Heikkila (2017, p.27–28) characterize policy conflict as a situation of entrenched divergence between policy positions of rival actors, who pursue their goals through institutional, coalitional, and/or contentious means. Policy conflict could thus vary in several ways: in *intensity*^4^ (from low to high such as transgression of established procedures and norms); in *orientation of activity* (from proactive policy advocacy to reactive mobilization^5^ against new policies); and in *timeframe* (from gradual struggles unfolding over many years to decades, to more rapid eruptions of discontent). Thus, policy conflict provides a useful overall frame although it is broader than backlash. Specific frameworks are needed for studying policy backlash because – as an intense, reactive, and abrupt form of policy conflict – it may differ qualitatively compared to moderate or low-intensity conflicts. Again, attention to domain-specific features is important for understanding why such reactions arise.

### Experiences from climate change policy

Climate policy is an important domain in which to study backlash. Climate policymaking is an immense challenge for governments globally to develop ambitious policy to meet the 2015 Paris Agreement goals (Falkner, 2016). As the perceived demands of climate policies grow, backlash risk could increase (IPCC, 2022; Patterson, 2023), especially given that hard/coercive policies are often thought to be needed for rapid decarbonization. Growing experiences of backlash highlight the need for comparative explanation.

Backlash to carbon pricing/taxation policies has been among the most dramatic. For example, the massive Yellow Vests Movement in France (2018–2019) was initially triggered by a scheduled carbon tax rise (Chamorel, 2019; Driscoll, 2023). In Australia, conservative opposition and social protests led to a national carbon pricing scheme being acrimoniously withdrawn in 2014 after just two years (Crowley, 2017). Canada has seen strong pushback at the provincial level in British Columbia in 2008–09 (Karapin, 2020) and in several other provinces in 2018–2019 who (unsuccessfully) challenged the constitutionality of national carbon pricing (Chalifour, 2019) and repealed provincial climate policy (Raymond, 2020). In Mexico, a surge in fuel prices partially caused by a carbon tax resulted in mass protests in 2017 (Caloca Lafont et al., 2020). And in the United States, attempts to establish an emissions trading scheme failed in 2010 following intense opposition among political representatives, media, and grassroots groups (Skocpol, 2013).

Other types of hard policy have also experienced backlash or related dynamics, including attacks on environmental regulations in the United States (e.g., power plants, vehicle emissions, oil and gas facilities) (Bomberg, 2021; Lin, 2022; Milman, 2025), and in Brazil (Toni & Feitosa Chaves, 2022). Several US states (e.g., Ohio, Arizona, Kansas, West Virginia) have also weakened or repealed renewable energy mandates and financial support (Breetz et al., 2018; Jang, 2022). While rollbacks do not necessarily constitute backlash (Sect. "Dependent variable"), strategic elite behavior may be linked to wider discontent among publics and organized groups. Other examples include a bottom-up referendum in California in 2010 attempting to suspend a state-wide emissions reduction target (Biber, 2013), and community opposition to a 2009 wind energy policy in Ontario, Canada leading to a moratorium in 2011 (Stokes, 2016).

Phase-outs may also experience backlash. In Germany, hostile negative reactions have occurred against diesel vehicle bans (Arning & Ziefle, 2020) and a proposed climate-related highway speed limit policy (Grünwald & Patterson, 2025). In the United States, the shutdown of coal industries and related job losses led to voter backlash (Egli et al., 2022). In Taiwan, an ambitious policy to phase out gasoline vehicles and scooters was suspended after less than two years due to strong pushback (from drivers, businesses, and industry) and broader electoral unpopularity (Liu & Chao, 2022). And in Sweden, a prohibition on new installations of wooden stoves was cancelled after two years following public protest over the curtailing of valued traditions and identities linked to wood burning in homes (Sahlberg et al., 2022).

These growing empirical experiences highlight the need for comparative explanations of backlash to climate policy. Yet, specific approaches for doing so are lacking, which risks idiosyncratic explanations and missed opportunity for broader insights.

## Studying backlash to climate policy

**Fig. 2:**
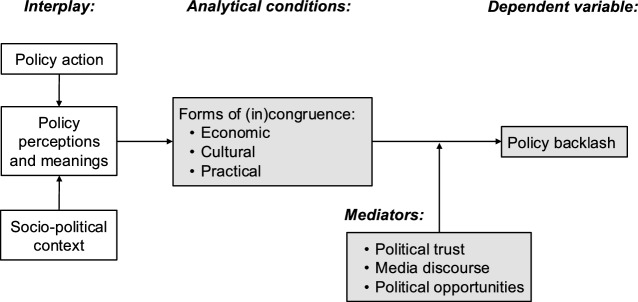
Framework to comparatively study the conditions for policy backlash

We develop a framework for the comparative analysis of the socio-political conditions under which backlash to climate policy occurs (Fig. 2), positing that backlash (the dependent variable, Sect. "Dependent variable"), is driven by incongruence between policy action and its socio-political context (Sect. "Conditions") and mediated by several other factors (Sect. "Mediators"). This combines diverse factors often studied separately such as economic costs, cultural grievances, and other impacts on people’s everyday lives (Bulkeley et al., 2016; Lockwood, 2018; Patterson, 2022). It also highlights the question of how a certain policy action becomes meaningful to target groups and wider audiences within a given time and place. In other words, it is not only objective policy impacts, but also what they come to mean, that is important. This helps draw together fragmented lines of thinking from comparative politics, policy, and sociology (e.g., following Hacker & Pierson, 2014, p.650; Steinberg et al., 2012; Weible & Heikkila, 2017).

### Dependent variable

**Fig. 3:**
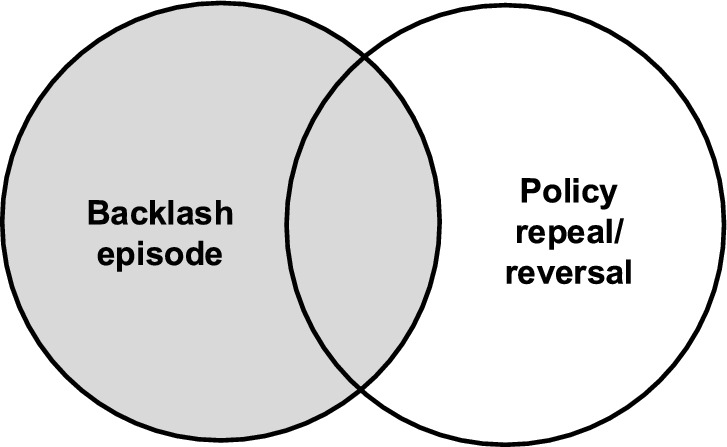
Analytical separation of a backlash episode and policy repeal/reversal that may or may not result

Backlash is an abrupt negative reaction attempting to reverse or remove a policy action. Importantly, it is distinct from policy repeal^6^ or reversal. Backlash is a reaction, an eruption of discontent against a policy action, but is not synonymous with the consequences for policy outputs^7^ (Fig. 3). Whether policy repeal/reversal results is an empirical question.^8^ This distinction aligns with our primary focus on an “episode” of policy conflict (Weible & Heikkila, 2017) – that is, the negative reaction itself. It also helps to separate policy backlash from longer-term strategic efforts by elites to roll back or dismantle policy. Although policy backlash and repeal/reversal might sometimes overlap, distinguishing them helps to focus on the reaction itself, regardless of policy change.

Empirical cases of policy backlash involve substantial contentious collective behavior in response to policy action. This could involve a variety of actors (mass publics, political elites, organized groups) and may even cut across typical political cleavages or trigger new groups to form. Mass publics are central actors in policy backlash because public discontent is the main force of backlash, even when mobilized into the public arena by elites.^9^ Public discontent may be channeled through institutional means (e.g., elections, party positions, court challenges) and non-institutional means (e.g., protests, strikes/blockades, public campaigns) (Patashnik, 2023; Patterson, 2023; Weible & Heikkila, 2017). Importantly, it may include hostile behaviors that transgress established procedures and norms (Alter & Zürn, 2020; Madsen et al., 2018). What matters is not necessarily the specific types of activities, but rather, the intensity of discontent conveyed. What is hostile and/or transgressive depends on norms in a given context, and can also change over time through shifts in what is considered extraordinary. It could relate to scale (e.g., amount of participation), character (e.g., fierceness of emotions, visibility), disruptiveness (e.g., property damage, hindrance, violence), symbolism (e.g., defection, antagonism), or rhetoric (e.g., aggression, vulgarity, threats). As a result, backlash threatens not only policy adoption/entrenchment but also the credibility and authority of policy proponents (following Alter & Zürn, 2020; Madsen et al., 2018; Patashnik, 2019; Patterson, 2023).

### Conditions

In our framework (Fig. 2), backlash occurs due to the incongruence between policy action and socio-political context. Incongruence is not solely objective but also comes from policy perceptions and meanings among target groups and wider audiences, whether rational (e.g., reflecting actual costs) or not (e.g., exaggerated costs). For example, partisan messaging can affect people’s perceptions of the costs and benefits of carbon pricing policy (Mildenberger et al., 2022). Moreover, perceptions and meanings can be reshaped during the policy process through heated debates (Stone, 2012). Identifying different dimensions of incongruence helps to understand sources of discontent underpinning backlash.

Our approach reflects a slightly different emphasis compared to Patashnik (2023). Patashnik focuses on explaining countermobilization, considering policy attributes along with the capacity and opportunity to mobilize. We focus on explaining the eruption of discontent, considering its diverse sources interpretively. A core strength of Patashnik’s approach is its generalizability as an explanatory framework for backlash politics across a wide range of policy domains. A core strength of our approach is its recognition of the context-embeddedness of policy action within a specific policy domain, motivated by a need to better understand how ambitious policy action can be advanced on a particular problem. Both approaches are complementary in seeking to disentangle the complex socio-political factors involved in policy backlash.

We contend that three key dimensions of incongruence can occur: economic, cultural, and practical (Table 1). In each dimension, a perceived mismatch can arise between the demands of policy action and the capacity of target groups to accommodate these demands. This could occur through direct impacts on target groups (e.g., economic, symbolic, or behavioral costs), differential impacts within target groups (e.g., how various costs are distributed), and/or wider impacts on society (e.g., further perceived consequences).

**Table 1:** Possible conditions for backlash to climate policy

Dimension	Condition	Explanation
Economic	Perception of excessive costs	Magnitude of costs seen as too high by target groups and wider audiences
	Perception of unfairness	Distribution of costs among different actors or groups seen as too unfair
	Misfit with broader economic circumstances	Policy action seen to worsen existing economic difficulties (e.g., economic insecurity, economic crisis)
Cultural	Clash with political beliefs	Policy action seen as being in contradiction with political beliefs (e.g., individual and market freedom, social protection)
	Threat to group identity	Policy action seen to threaten social identity, especially if reinforcing existing threats (e.g., livelihood, community, social status)
	Misfit with broader cultural values	Policy action seen as antagonistic to broader cultural context (e.g., signifying undesirable progressive values)
Practical	Discomfort with new practices	Changes required by policy action seen as uncomfortable or disruptive in practice
	Unfeasible /impossible expectations	Changes required by policy action seen as unfeasible or impossible to meet for some target groups (e.g., lack of infrastructure)
	Clash with other areas of life	Changes required by policy action seen to clash with other valued practices (e.g., responsibilities and obligations)

#### Economic

Economic aspects are central to climate policy debates (Harrison et al., 2010; Jordan et al., 2010). Economic incongruence involves a logic of rationality and refers to a significant perceived mismatch between a policy’s economic impacts (e.g., costs and benefits) and the preferences and interests of target groups and wider audiences.

*Perception of excessive costs* can arise from both magnitude and visibility (Patashnik, 2019, p.51–52; Pierson, 1993, p.620), and may be shaped by both elite cues and peoples’ own experiences and/or beliefs (Patashnik, 2023, p.21–22). Although climate policy may involve compensation through revenue recycling, assigned investment, or tax neutrality (Drews & van den Bergh, 2016), this does not guarantee acceptance (e.g., Crowley, 2017; Raymond, 2020). Visible costs provide more opportunity to be challenged than opaque costs (Finnegan, 2022). Elites can also strategically construct policy gains and losses to, for example, stoke anger (Patashnik, 2019; Skocpol, 2013) or fears over job losses or other changes (Iskander & Lowe, 2020).

*Perception of unfairness* can arise when climate policy is seen to impact different groups differently, particularly in contexts of pre-existing social or economic inequality. This perception is a key factor influencing public support for climate policy in general (Bergquist et al., 2022; Dechezleprêtre et al., 2022). Perceptions of fairness might vary by group (e.g., depending on socioeconomic status or geography). Conversely, a sense of unfairness could stem from existing benefits being withdrawn or from “undeserving” others being allocated benefits (e.g., subsidies) (Patashnik, 2023, p.24–25).

*Misfit with broader economic circumstances* can arise when wider economic crises undermine the priority of climate policy. Economic crises can create pressure to roll back climate policy (Prontera, 2021) and the political costs of climate policy can be higher when fuel prices are high (Furceri et al., 2021). More broadly, “voters penalize parties associated with post-material issues or those with long-run payoffs during economic downturns” (Abou-Chadi & Kayser, 2017, p.201). Rising grievances during economic downturns may also increase protest activity (Kern et al., 2015). Hence, unfavorable economic circumstances might lead to climate policy becoming unrepresentative of “preferences and priorities of voters” (Patashnik, 2023, p.26).

#### Cultural

Cultural aspects of climate policy are increasingly recognized due to cultural resistance (Brown & Spiegel, 2019; Patterson, 2022) and interlinkages with right-wing populism (Lockwood, 2018). Cultural incongruence involves a logic of appropriateness and refers to a significant perceived mismatch between a policy’s cultural impacts (e.g., symbols, values signified) and the norms and values of target groups and wider audiences.

*Clash with political ideology or beliefs* can arise when climate policy is seen as contravening broader political stances, such as priority for individual and market freedoms (Skocpol, 2013) and climate denial (Crowley, 2017; Raymond, 2020). These factors could amplify incongruence through strategic party differentiation (Patashnik, 2019) and ideological polarization (McCright & Dunlap, 2011). For example, rapid partisan polarization occurred over the proposed Green New Deal in the United States in 2019 (Gustafson et al., 2019). Yet, such patterns are not always straightforward. For example, conservative voting preferences are not always associated with renewable energy opposition (Bessette & Mills, 2021). This suggests that political ideology may interact with other conditions or vary in influence.

*Threat to group identity* can arise when climate policy threatens social identities. This can include social identities established endogenously by past policies (Patashnik, 2019, p.54) or exogenous to policy (e.g., linked to economic or geographical marginalization, place identification, or socio-economic status/class). For example, non-urban communities might perceive climate policy as threatening to their way of life, especially if they already feel portrayed negatively. Climate policy may be seen as threatening livelihood identities (e.g., linked to fossil fuel production) through negative portrayals of these activities, and could also resonate with a sense of national identity involving pride in fossil fuels (Kuchler & Bridge, 2018). Norms of masculinity tied to fossil fuels may also exacerbate the perceived threat posed by climate policy (Daggett, 2018). Identity threats can be amplified by affective polarization (Iyengar et al., 2019) and right-wing populism (Lockwood, 2018).

*Misfit with broader cultural values* can arise when climate policy becomes embroiled in wider cultural discontent. For example, anti-cosmopolitan pushback against socio-cultural change (Norris & Inglehart, 2019) might also affect climate policy. In this vein, Lockwood (2018) questions whether right-wing populist opposition to climate action is partly such “collateral damage”. From another angle, the conjunction of cultural and economic factors, such as subjective social status decline and loss of dignity (Gidron & Hall, 2017) can create resentment. This could condition responses to climate policy, for example, if those lacking cultural and economic capital perceive climate policy as imposing deprivation or reflecting elite status-seeking (Laidley, 2013).

#### Practical

The practical implications of climate policy action within real-world settings is another important dimension. Climate policy intersects with peoples’ everyday practices, where possibilities for change are circumscribed by available infrastructure, technology, and circumstances (Bulkeley et al., 2016; Weaver, 2014). This involves a logic of embeddedness and refers to a significant perceived mismatch between policy expectations for behavior change and people’s practical ability to meet them.

*Discomfort with new practices* can arise when climate policy is seen to demand undue discomfort or disruption. This could include expectations to adopt new technologies that are unfamiliar or believed to perform poorly (e.g., home heating/cooling), to use transport perceived as uncomfortable or difficult (e.g., patchy or unreliable transport infrastructure), or undergo intrusive measures in homes (Devenish & Lockwood, 2024). Discomfort can also come from being compelled to abandon valued practices such as use of wood stoves (Sahlberg et al., 2022) or fast driving (Grünwald & Patterson, 2025).

*Unfeasible/impossible expectations* can arise when climate policy creates expectations that not all affected people can meet. Weaver (2014) argues that target groups may “lack the resources that they need to adapt to a policy, even if they want to comply” (p.246), because compliance may require not only financial resources but also health, individual and social capital, and access to infrastructure (p.249). For example, switching mobility modes is often not possible for many people such as those on lower incomes or those unequally served by supporting infrastructure (e.g., public transport, electric vehicles) (Liu & Chao, 2022). Furthermore, people’s ability to make major changes in their homes can vary greatly (e.g., due to financial circumstances, or technology availability). Everyday practices can also be ‘locked in’ and hard to change in isolation due to reinforcing technological, institutional, and historical aspects (Seto et al., 2016), creating multiple compliance barriers for people (Weaver, 2014, p.250).

*Clash with other areas of life* can arise when climate policy negatively impacts other valued practices. For example, climate policy might conflict with the desire for comfort (e.g., keeping a poorly insulated house warm), meaningful work (e.g., expertise in certain professions), or other aspirations (e.g., leisure, travel, care) (Bulkeley et al., 2016). Moreover, incentives may be perceived differently within target groups leading to different effects (Weaver, 2014, p.243). Therefore, discontent with climate policy might arise not only because of conscious opposition but also due to implicit contradictions with other aspects of people’s lives (Bulkeley et al., 2016).

### Mediators

Several mediators may also influence the extent to which perceived incongruence spreads. These mediators do not create incongruence on their own but could influence its intensity. They are directionally non-determinative (i.e., they could affect incongruence in different ways) and are not necessary. Three important mediators likely to be important based on empirical experiences of conflict over climate policy are:

- *Political trust*: This can moderate whether incongruence is accepted and how opponents apportion blame. Low trust may foster the spread of discontent because people lack faith in policymakers’ intentions or attention to peoples’ concerns, or are skeptical about promised future benefits (e.g., Driscoll, 2023; Ewald et al., 2022). High trust may foster belief in policymakers’ intentions and acceptance that concerns are considered, even if people disagree with the policy itself.
- *Media discourse:* This can moderate the spread of discontent by contributing to the salience and meaning of climate policy. For example, media discourse can amplify elite cues (Hacker & Pierson, 2019, 17), reinforce prior beliefs (Bolin & Hamilton, 2018), and activate latent attitudes (Bonikowski, 2017). Negative media discourse might help discontent over policy action to spread, while positive media discourse might dampen its spread. Yet, the role of media discourse is ambiguous in contemporary mediatized politics (Hjarvard, 2013) and it could sometimes be an actor in backlash. We suggest addressing this empirically, beginning by considering it as a mediator.
- *Political opportunities:* This can moderate the chance of discontent coalescing. Opportunities could include proximity to elections (Weible & Heikkila, 2017), competition among elites (Patashnik, 2023, p.29) keeping conflict open, external shocks providing a focus for discontent (Nohrstedt & Parker, 2024), or new networks among heterogeneous actors sparked by common concerns (McAdam et al., 2001, p.26).

## Illustrative cases

We apply our framework to three illustrative cases of backlash to climate policy in Canada, France, and Mexico (Table 2). These are all cases of backlash to national carbon pricing policy: in Canada, concerning a new introduced federal carbon pricing scheme introduced, and in France and Mexico concerning an increase in existing carbon prices. All three cases share similarities including overall policy type (carbon pricing), target groups (consumers and industry), and level of ambition (intention to influence behavior with design elements to reduce policy burden). Yet, the cases are similar/different in other ways. All are geographically large countries with dispersed populations, two are federal (Canada, Mexico) and one is unitary (France), and two involve substantial socioeconomic disparities (especially Mexico, but also France to a lesser extent). Backlash manifested differently between the cases: 1) through province-level pushback and constitutional challenges (Canada), 2) massive public protests (France), and 3) many dispersed public protests (Mexico). Policy outcomes also varied: the policy remained in place in Canada, the tax was frozen in France, and the policy was moderated in Mexico. Therefore, while the cases are broadly consistent, they also jointly exhibit moderate diversity that helps support plausibility probing of our approach.

**Table 2:** Overview of the three illustrative cases

Case attributes	Case 1: Challenges against the Greenhouse Gas Pollution Pricing Act in Canada (2018–2021)	Case 2: Protests against carbon tax rise in France (2018–2019)	Case 3: Protests against carbon tax rise in Mexico (2017)
Policy action	Federal carbon pricing scheme introduced in 2018	Scheduled increase in national carbon price	Increase in carbon tax in 2017
Design features	Separate industry and consumer components Province/ territory differentiation allowed if stringency maintained Revenue recycling; rebates; industry assistance	Progressive annual increase in carbon tax since 2014 Most revenue goes to general budget rather than decarbonization specifically	Federal carbon tax introduced in 2014 Embedded within energy sector liberalization reform Exemptions for manufacturing fuels Revenue goes to general budget rather than decarbonization specifically
Background	Contention over prior climate inaction Intense negotiation between federal and province/ territory levels	Growing socio-economic and regional inequalities Distrust and antipathy towards elites (politicians, media, intellectual)	High level of economic vulnerability and social disparity Ideological divides
Backlash	Several provinces mobilise against scheme; Premiers opposing scheme are elected Constitutional challenge and other attacks on climate policy by some provinces	Massive wave of sustained popular protests across country over many months Carbon tax frozen and new initiatives begun to develop acceptable climate policy	Wave of protests against the policy and government Subsequent leftward change in political leadership Moderation of policy ambition

### Canada: Greenhouse Gas Pollution Pricing Act

The first case concerns the 2018 *Greenhouse Gas Pollution Pricing Act* (GGPPA) which sets Canada’s national carbon price. It comprises a fuel charge (carbon tax) applied to consumers through retail fuel prices and an Output-Based Pricing System (OBPS) that sets emissions efficiency benchmarks (ECCC, 2024a, 2024b; Harrison, 2023, p.77). The carbon price is then applied to industrial facilities that exceed their emissions benchmark. The scheme has two features intended to insulate it from backlash: i) revenue recycling through industry assistance and consumer dividends (ECCC, 2024c), and ii) its “benchmark and backstop” design (ECCC, 2017; Mascher, 2018) that means it only applies to provinces if their own prices are insufficient or they voluntarily opt in (ECCC, 2024a). The benchmark price ratchets over time.^10^ These features aim to minimize cost imposition and allow provincial flexibility. Despite some criticism over design and coverage,^11^ the scheme is considered relatively ambitious (Parry, 2021) and has survived two federal elections (Bakx, 2019; Ivison, 2018; Raymond, 2020, p.1137). This is significant given Canada’s regionally diverse and carbon-intensive economy (Harrison, 2019), and its history of political battles over carbon pricing.

The national context in which the GGPPA arose was politically divisive and regionally polarized on climate change (Leach et al., 2021). Previous policy efforts had failed (Carter et al., 2017, p.63; Harrison, 2023, p.72; Macdonald, 2020, p.4), notably during the 2008 federal election where conservatives successfully weaponized a proposed carbon tax by the Liberals’ as an economic threat (CBC News, 2008; Harrison, 2012, p.397; Macneil, 2020, p.357), and there was a long period of inaction afterwards (Macdonald, 2020; C. Meyer, 2019; Partington, 2013). Nonetheless, Justin Trudeau (Liberal) successfully campaigned on climate policy in 2015, leveraging public dissatisfaction with prior government inaction (KAIROS, 2010; McCarthy & Blackwell, 2013), limited conservative opposition, expert advocacy (e.g., Ecofiscal Commission, 2014) and international support for climate policy under the Paris Agreement. A rare domestic alignment with provincial governments led to a “Pan-Canadian” approach to carbon pricing emphasizing sub-national flexibility (Harrison, 2023, p.74; Macneil, 2020, p.354; MacNeil & Paterson, 2018, p.380).

Nonetheless, backlash against the GGPPA soon arose, spearheaded by conservative politicians and concentrated among conservative voters and provinces. Shortly after the GGPPA was legislated, federal-provincial alignment disintegrated (Harrison, 2019). Ontario elected a populist conservative government in 2018 that immediately repealed Ontario’s cap and trade scheme and withdrew from an ETS linkage initiative with California (Raymond, 2020). In 2019, Alberta’s incoming conservative government similarly rescinded Alberta’s fuel charge (Harrison, 2023, p.77). These provinces joined Saskatchewan (which had consistently opposed carbon pricing) to vocally campaign against the federal carbon pricing scheme (Macneil, 2020, p.356). A Yellow Vests Canada movement, inspired by the Yellow Vests in France, arose in late 2018, with local and federal elites often in attendance at rallies and truck convoys (e.g., Baxter, 2019). While on-ground protests were minimally disruptive (De Cillia & McCurdy, 2020), online rhetoric was hostile (Tewksbury, 2021, p.940). Several provinces (Ontario, Alberta, Saskatchewan) eventually challenged the GGPPA’s constitutionality (Chalifour, 2019), although the Supreme Court upheld the GGPPA’s constitutionality in 2021 (Supreme Court of Canada, 2021). However, conservative- and elite-led opposition continues, even though less intensively (Boynton, 2024; Macneil, 2020, p.357). Canada’s national conservative party have pledged to repeal the fuel charge if elected in 2025, in a context where public opinion on climate policy has become increasingly partisan over time (Harrison, 2023, p.69; Lachapelle & Borick, 2022, p.11).

Several economic, cultural, and practical conditions underpinned this backlash. Despite revenue recycling being designed to mitigate cost impacts, the *perception of excessive costs* was central. Conservative elites emphasized and inflated costs, consistently labelling the GGPPA as a “job-killing carbon tax” (Bakx, 2019). The federal opposition leader claimed that the GGPPA would increase the costs of “…everything from driving your kids to school, to heating your home, to your groceries” (Scheer, 2019). In Alberta, opposition leader Jason Kenney “…relentlessly attack[ed] the carbon price as effectively the root of all of Alberta’s problems” (Macneil, 2020, p.357). The new Ford government in Ontario blamed the GGPPA for various economic pressures, stating that it “…makes life more unaffordable and hits the wallets of Ontario families” (CBC News, 2020) and “…drain[s] resources from our police, firefighters, and paramedics” (Ford, 2019). He also infamously mandated stickers on gas pumps emphasizing the costs of the carbon price, which was criticized as misleading due to omission of rebates (Jeffords, 2019; McIntosh, 2019) and later deemed unconstitutional for being blatantly political (CBC News, 2020). Elite rhetoric likely swayed public perceptions of costs. Mildenberger et al. (2022) found that Canadians persistently underestimated GGPPA rebates and that providing more information decreased support (especially among conservatives), and Lépissier et al. (2022) found that people’s perceptions of costs better explained carbon tax opposition than changes in actual costs. Macneil (2020, p.357) views Kenney’s 2019 election win as an example of weaponized anti-carbon pricing sentiment, although this was less clear for Ford in Ontario in 2018 (Lachapelle & Kiss, 2019). Perceptions of excessive costs continue to fuel conservative opposition to the GGPPA, where the (perceived) steep trajectory of annual price increases remains in focus (Hopper, 2023).

*Perceptions of unfairness* arose from regional differences and the GGPPA’s complex implementation. Despite province-specific revenue returns, some provinces felt disproportionately burdened by the GGPPA (Carter, 2020; Harrison, 2023, p.69; Macdonald, 2020, p.13). Canada’s provinces and territories have different resource bases, manufacturing sectors, and energy systems, leading to large differences in carbon intensity (Harrison, 2023). The GGPPA was seen as a threat to economic competitiveness in carbon-intensive provinces, creating concern about concentrated job losses (Macdonald, 2020). Shifting benchmarks and a patchwork of provincial policies have also hindered the communication of rebates, allowing misinformation and claims of unfairness to spread (Mildenberger et al., 2022; Rabson, 2024).

A *clash with political beliefs* was also central. Partisanship has long been a predictor of Canadians’ climate policy preferences, underpinned by continued divisiveness on climate change (Harrison, 2023, p.69; Lachapelle & Borick, 2022, p.7). In 2020, less than 30% of conservatives attributed climate change primarily to human causes, compared to over 70% of progressive party supporters (Lachapelle & Borick, 2022, p.9). Puelma Touzel and Lachapelle (2024, p.10–14) found that conservatives opposed the GGPPA through a “more well-worn, coherent ideology” than that of supporters, suggesting it was easier for conservative elites to galvanize opposition than for the government to garner support.

Related *threats to group identity* also arose. Conservative national identity remains tied to the idea that Canada is an ‘energy superpower’, underpinning resistance to climate policy (MacNeil & Paterson, 2018, p.382). In parallel, strong beliefs about provincial sovereignty intersected with opposition to the GGPPA and even cut across political parties. Carbon-intensive and traditionally conservative provinces (notably Alberta and Saskatchewan) asserted that the GGPPA infringed on their provincial right to extract natural resources (Raymond, 2020, p.1136). Provincial elites’ rhetoric echoed that of their federal counterparts, also emphasizing provincial autonomy^12^ (Baxter, 2019). The constitutional challenge by Alberta, Ontario, and Saskatchewan exemplified this. However, even the pro-climate action province of Quebec intervened in support of the constitutional challenge on the basis of protecting provincial jurisdiction (CBC News, 2019). Thus, partisan polarization and group identities likely facilitated backlash.

Practically, *unfeasible expectations* and *discomfort with new practices* also contributed. The most volatile protests occurred in carbon-intensive provinces. Even with revenue returns negating costs, some regions faced greater industrial and lifestyle impacts than others (Harrison, 2019). In 2018, Jason Kenney (then opposition leader) claimed that two-thirds of Albertans opposed the GGPPA, not because they were indifferent to climate action, but because: “punishing consumers for living normal lives in a cold northern climate and an advanced economy is not a responsible environmental policy” (Kenney, 2018). Lépissier et al. (2022) observed stronger opposition to carbon pricing among car commuters and rural residents, suggesting concerns over lifestyle changes.

Other factors also mediated backlash. First, *media discourse* contributed to spreading discontent. Canada’s media is typically considered nonpartisan, but leans somewhat ideologically right on economic issues (Thibault et al., 2020), which may have led to an emphasis on costs. Media also quickly labelled Ford’s victory in Ontario a “death sentence” (Macneil, 2020, p.357) for the Pan-Canadian approach, despite other factors being more significant predictors of support for Ford (Lachapelle & Kiss, 2019). Second, *political opportunities* influenced both support and opposition. Fortuitous federal-provincial alignment initially helped, but subsequent provincial elections enabled certain provinces to become strong opponents. Despite the provincial opposition coalition, the federal Conservative’s anti-tax campaigns failed in two national elections. At the same time, climate-related wildfires and school student strikes bolstered support for climate action in the 2019 federal election (Bricker, 2019). Nonetheless, by 2023, polls showed that most Canadians wanted carbon pricing reduced or removed (Hopper, 2023), and the Conservative opposition continued to promise to repeal carbon pricing. Sentiment remained ideologically polarized, however, with the majority (60–70%) of progressive-leaning voters continuing to support carbon pricing in February 2025 (Keating & Lempriere, 2025). A crisis of public trust in Trudeau and broader public economic concerns (Leichnitz, 2025) provided new opportunities for conservatives to leverage public opposition to carbon pricing as a political wedge in the lead up to the 2025 election (Keslter-D’Amours, 2025). To neutralize this risk, the incoming Liberals leader Mark Carney declared the consumer charge as untenable and functionally ceased its implementation (by setting the price to zero) from April 2025 (The Canadian Press, 2025) with public opinion divided in response (Tindall et al., 2025), although the industrial scheme remains in force.

### France: carbon tax and the yellow vests

The second case is the Yellow Vests Movement (YVM), a nationwide protest movement that emerged in response to an impending carbon tax increase in France (Driscoll, 2023; Martin & Islar, 2021). This tax had already been in place and had risen progressively since 2014. Its aim was to encourage less driving, to transition away from diesel vehicles by gradually aligning diesel and gasoline prices, and to enable the reduction of taxes on other products, work, or income (Durand, 2018). Despite its incremental design, the impending price increase unexpectedly sparked a massive spontaneous eruption of discontent across the country in November 2018. Protests persisted for several months and became the largest and longest-lasting protest movement in France for many decades (Chamorel, 2019; Driscoll, 2023).

France’s prior experiences with carbon taxes had been mixed. Following social and industrial opposition, earlier attempts to introduce a carbon tax (2000, 2009) were rejected by the Constitutional Council due to a breach of equality before tax (Durand, 2018; Rocamora, 2017). In 2014, a “carbon component” was incorporated into existing energy taxes, starting at 7 €/tCO2 (Criqui et al., 2019) to circumvent censorship by the Constitutional Council (Criqui et al., 2019; Durand, 2018; OECD, 2019). The low starting price and a coincidental decline in world oil prices meant that it was largely unnoticed at first (Criqui et al., 2019; Durand, 2018). However, it increased regularly. By 2018, it had risen from 7 €/tCO2 to 44.60 €/tCO2 (Durand, 2018). In that same year, the French Assembly voted to double it to 86.2€/tCO2 by the end of Macron’s presidency (Douenne & Fabre, 2022; Durand, 2018). This was projected to add 6.5 eurocents per liter to diesel prices at a time when almost 60% of private vehicles in France were diesel (Martin & Islar, 2021, p.602).

In response, an online petition calling for “a drop in fuel prices at the pump” (cited in Driscoll, 2023, p.7), launched in May 2018, gained increasing momentum. Roadblocks and protests began on 17 November, with 290,000 protesters gathering across France (Driscoll, 2023; Martin & Islar, 2021). Weekly protests followed (Regan, 2019), involving both confrontational mass protests in many cities and symbolic actions (e.g., wearing the yellow vest and occupying roundabouts) (Wahnich, 2020). The movement united people from rural, peri-urban, and urban regions and was “diverse in its political leanings” (Grossman, 2019). Although “working and lower middle classes [were] over-represented”, some upper-middle class participants also joined (Dormagen et al., 2022, p.444). Support for the movement grew as focus shifted to much wider and longstanding grievances (e.g., social inequality, difficulties for many people in making ends meet, resentment towards elites (Driscoll, 2023; Grossman & Mayer, 2023; Martin & Islar, 2021). By the end of November, over 80% of French people supported the YVM (Lazar, 2019).

The movement had immediate and long-lasting effects. Less than three weeks after the protests began, Prime Minister Edouard Philippe announced that the tax increases would be frozen for six months to “bring calm and serenity back to [France]” and “enable real dialogue” on the protesters’ concerns (Al Jazeera, 2018a). However, protests continued (Al Jazeera, 2018b). The next day, the government abandoned the tax increase altogether (Willsher, 2018), confronting the French government with its worst political crisis in decades (Chamorel, 2019). Yet, the YVM persisted, now embodying much more deep-seated grievances against the political system and institutions (Girerd et al., 2020; Lazar, 2019). However, public support and the number of participants in the YVM eventually declined (Grossman & Mayer, 2023).

Several economic, cultural, and practical conditions underpinned this policy backlash. Economically, the *perception of excessive costs* was strong, especially among rural, peri-urban, and working-class people already experiencing cost-of-living difficulties (Martin & Islar, 2021). According to Martin and Islar (2021, p.603), 65% of YVM participants were “having difficulties making ends meet with a standard of living amounted at €1.486/month, compared to a national average of €1.777”. Against this crisis of purchasing power (Sananes & Bedeau, 2019), increasing fuel prices were considered excessive: “The carbon tax will soon rise to 10, 15, 20, 50, 70 euros per month […]. That’s the cost to fill up my car or buy my groceries!” (cited in Driscoll, 2023, p.15). This was linked to a sense of *misfit with broader economic circumstances*: the tax largely went unnoticed until 2017 due to low world oil prices, but the rise in oil prices combined with the increase in the carbon tax made it more visible by the end of 2018 (Durand, 2018). Additionally, the annual increase in the tax coincided with amendments designed to converge the prices of gasoline and diesel, resulting in a “sharp and rapid increase in [energy taxes]” (Durand, 2018).

Adding to this was a strong *perception of unfairness*. The carbon tax increase was seen as a demand for sacrifice by working-class people who could least afford it, when taxes on the wealthy were being simultaneously reduced and fuel taxes were already high relative to other European countries (Chamorel, 2019; Jetten et al., 2020). Rural and peri-urban people also felt disproportionately affected because of their dependency on private vehicles (including high rates of diesel vehicle ownership) and a lack of public transport (Driscoll, 2021). Practically, a sense of *unfeasible or impossible expectations* was also substantial. “The population has suffered for years […] and people continue to suffer” (cited in Driscoll, 2023, p.15). A seemingly benign increase in carbon taxes became “the straw that broke the camel’s back” (cited in Driscoll, 2023, p.15).

Culturally, a sense of *threat to group identity* was pronounced, including a feeling of loss of dignity from cost burdens (Grossman, 2019) that compounded a longer-term sense of eroding socio-cultural capital, especially in peripheral regions (Bourdin & Torre, 2023). The Yellow Vests were united not only by social and economic grievances but also by a strong resentment towards elites and elite institutions (Dormagen et al., 2022). As the carbon tax was seen to benefit these elites, there was also a perceived *misfit with broader cultural values*. Protestors portrayed the carbon tax as contravening egalitarianism (Chamorel, 2019) and as “a direct threat to the French national identity and … French values” (Jetten et al., 2020, p.2). This places the YVM in a much longer history of class struggle in France, particularly between “the people” and “the privileged” (Grunberg, 2019).

Other factors contributed to mediating backlash. In particular, *political trust* had been declining since 2010 (Grossman, 2019, p.23). By 2019, the Barometer of Political Confidence recorded the lowest levels of trust in the president, the government, the National Assembly, and the Senate in the ten years the barometer had existed (Cautrès, 2019, p.2). With 74% of people believing that the state only acted in the interests of a small minority, it concluded that “the way the French look at politics and the judgement they pass politicians is extremely gloomy”^13^ (Cautrès, 2019, p.1). In this light, the YVM was not only a response to the carbon tax but also a “response to an intense crisis of political trust” exacerbated under President Macron (Grossman, 2019, p.30). This decline in political trust aggravated the social and economic cleavages that were made more visible through the carbon tax.

Declining trust in traditional *media discourse* (Cautrès, 2019; Chamorel, 2019) was another important mediator. Media coverage of the YVM, which accounted for 20% of televised news between November 2018 and March 2019, was “unprecedented” (Poels & Lefort, 2019). However, the media’s role was mixed. Initially, media coverage highlighted the social and economic claims of protestors (Poels & Lefort, 2019), although political figures rather than protestors themselves were “most often invited to comment on the movement”. However, trust in traditional media fell sharply due to accusations they were “sensationalis[ing] events and [interviewing] polarised or extreme witnesses” (Newman et al., 2019, p.83). Reporters even “faced verbal and physical attacks, with a number thrown to the ground and even mugged” (Newman et al., 2019, p.83). Some people turned to alternative news sources (e.g., RT France, Sputnik) and social media, which played throughout the YVM (Joux, 2019). Hence, media discourse both aggravated perceptions of unfairness and helped to make it more visible.

### Mexico: carbon tax and energy sector reform

The third case concerns a federal carbon tax (*Impuesto Especial sobre Producción y Servicios* – IEPS) introduced by President Peña Nieto (centre-right Institutional Revolutionary Party—PRI) in 2014 (Mehling & Dimantchev, 2017). While its ostensible aim was emissions reduction (Garcia et al., 2021), the tax also aimed to raise general revenue to reduce economic dependence on oil revenue (Belausteguigoitia et al., 2022, p.138; Grunstein, 2017). Policy designers analyzed social impacts and public support for the tax (Belausteguigoitia et al., 2022), incorporating features aimed at mitigating backlash from the public and industry. It was designed to minimize public attention through being introduced as part of a special tax on products and services and embedded within wider energy sector liberalization reforms (Black et al., 2021; Mehling & Dimantchev, 2017; Skovgaard & Ferrari, 2023). It was also framed publicly as a climate change rather than revenue raising instrument because environmental protection was more popular than “taking people’s money” (Skovgaard & Ferrari, 2023).

To ease cost impacts, it was introduced at a low rate (starting at USD$3.50 tCO₂ equivalent on average – or about 3% of the fuel price in 2014), and was set to increase minimally and gradually (Arlinghaus & van Dender, 2017; Barragán-Beaud et al., 2018; Mehling & Dimantchev, 2017). Companies were given the opportunity to reduce their carbon tax liabilities by purchasing carbon credits from UN-certified projects (Mehling & Dimantchev, 2017) and tax rates were to be proportional to each fuel’s emissions potential (Belausteguigoitia et al., 2022). Following legislative negotiations, natural gas and non-combustion fossil fuels used for manufacturing were effectively exempted through a static zero price, and the rate for coal was set much lower than for other fuels (Arlinghaus & van Dender, 2017; Barragán-Beaud et al., 2018, p.288; Belausteguigoitia et al., 2022, p.143). Overall, these features and compromises around industry impacts meant the carbon tax was very modest in terms of climate ambition, especially initially, and it also fell short of its original revenue-raising potential (Belausteguigoitia et al., 2022). Nevertheless, the tax was successfully legislated and operated^14^ without major pushback from 2014–2017.

In early 2017, mass protests erupted in response to the most recent tax adjustment with a ferocity and breadth that surprised commentators (and likely also the government) (Velador & Herrera, 2021). Backlash spread nationwide via multiple forms, including road blockades, gas station occupations, demonstrations,^15^ strikes, official complaints, looting and vandalism of businesses, and disobedience against police and law enforcement (Gutiérrez, 2020; Ríos, 2017). Numerous groups called for action to mitigate energy price increases, including the Employers' Confederation of the Mexican Republic, the Conference of the Mexican Episcopate, peasant organizations, unions, opposition parties,^16^ and even political parties and some NGOs that had previously supported the IEPS (AP, 2017b; Grunstein, 2017; Machaca, 2017). Interest groups who remained supportive (environmental NGOs, some enterprises) were not strong or organized enough to defend these demands (Belausteguigoitia et al., 2022, p.146).

While triggered by the fuel price rise, the protestors’ demands also rapidly widened to a variety of additional government positions and policies (e.g., demanding a wholesale budget review, rejecting the Trans-Pacific Partnership, repealing the privatization of water, eliminating taxes, removing car registrations, and stopping unemployment) (Caloca Lafont et al., 2020. p.9; Reyes & Abraham, 2017, p.11). In response, the government introduced additional measures to prevent price increases in essential products and avoid cuts in social programs (Ahrens, 2017) and then backed away from the scheduled tax increase altogether (Grunstein, 2017). Despite these concessions, criticism of how the government handled the situation continued throughout the 2017–2018 presidential campaign. Opposition candidate Andrés Manuel López Obrador (PRD) leveraged the issue against the PRI government, eventually winning the presidential election.

Economic and cultural conditions were especially important to this backlash. Economically, a *perception of excessive costs* arose in response to an abrupt surge in fuel prices (≥ 20%) at the beginning of 2017, with 59% of survey respondents saying their household was greatly affected (AP, 2017a). Although the IEPS was not the only driver of this price spike (the removal of energy subsidies, privatization of energy markets, and international energy market volatility also contributed), people could not differentiate between these factors (Paullier, 2017). The surge in prices was immediately attributed to the entire reform package and the president in particular (Caloca Lafont et al., 2020). The government launched an information campaign to explain the reform, including the place of the carbon tax (e.g., Reza, 2017), but it had little effect on cost perceptions (Paullier, 2017).

*Perception of unfairness* also arose around the IEPS disproportionately affecting low-income households and small businesses. Low-income households were more vulnerable to fuel price increases and relied more on carbon-intensive goods and services (e.g., older, less fuel-efficient vehicles, fossil fuels for electricity and heating) (Renner, 2018). Practically, this also created a sense among these groups that the policy involved *unfeasible or impossible expectations*. Additionally, small and medium-sized businesses with low profit margins and communities whose jobs depended on emissions-intensive industries perceived the policy as unfair (Garcia et al., 2021). A lack of transparency in government revenue flows exacerbated public perceptions that state spending was being bolstered by carbon tax revenues rather than other means (such as oil revenues or higher wealth taxes) (Grunstein, 2017). A sense of *misfit with broader economic circumstances* was also pronounced. Over 40% of Mexico’s population still lived in poverty (World Bank, 2021) and economic insecurity was widespread. In this context, the government’s perceived mismanagement of the issue led to calls for “…a more dignified and fair life for all”^17^ (Reyes & Abraham, 2017, p.11).

Culturally, the carbon tax also created a *clash with political beliefs* among those already skeptical of the government’s policies. Specifically, the significant involvement of the World Bank, International Monetary Fund and other foreign development agencies fueled perceptions that the IEPS was part of a neoliberal project violating Mexico's national interests, with anti-neoliberal discourse widespread (Caloca Lafont et al., 2020; Zuart Garduño & Herrán Aguirre, 2021). *Misfit with broader cultural values* was also significant due to the role of the fossil fuel industry in Mexico’s national imaginary. For instance, Oil Expropriation Day celebrates the nationalization of the country’s oil fields and the state-owned and operated petroleum company PEMEX is a symbol of pride (Garcia et al., 2021). These deeply held values have contributed to resistance against oil sector reforms in the past (Arlinghaus & van Dender, 2017; Caloca Lafont et al., 2020) and similarly contributed to resistance against the IEPS.

Several other factors also contributed to mediating backlash. *Political trust* was important due to low public trust in state institutions. Public trust decreased from 2000–2018 due to perceptions of government corruption and increasing inequality (Zuart Garduño & Herrán Aguirre, 2021, p.14). The limited transparency of revenue flows from the IEPS exacerbated doubts that the revenue was being properly spent (Grunstein, 2017). Moreover, the price spike was seen as a failed promise. For example, Machaca (2017, p.29) reflected that: “we were told that the increases in the prices of gasoline, electricity and gas would end and even decrease. … Today, everything is seen as a terrible lie.”.^18^ Grunstein (2017) also noted that: “Even NGOs that had previously criticized gas subsidies said that the higher prices were unjustified. Some accused the government of theft and corruption”. Some citizens even alleged on social media that eruptions of violence were being procured by the government to discredit otherwise peaceful protests (Caloca Lafont et al., 2020, p.9).

*Media discourse,* especially through social media, was also important. Analysis of Twitter showed criticism of price increases uniting opinion leaders, politicians, and youth across the political spectrum (Caloca Lafont et al., 2020). Yet, social media also fueled backlash through, for example, premeditated looting (Velador & Herrera, 2021) and fake accounts encouraging vandalism (Woody, 2017). Misinformation was even spread by traditional media, although these instances were rare (Caloca Lafont et al., 2020, p.10).

*Political opportunity* was less important due to the bottom-up nature of backlash. However, the proximity to a presidential election (held every six years) was important. The opposition party (PRD), led by López Obrador, was opposed to the carbon tax increase, framing the associated price surge as a national emergency and warning that it risked triggering social unrest (AP, 2017b). Some PRD members encouraged peaceful protests to stop the fuel price rise, suggesting an awareness of its potential political benefits (AP, 2017b). During the protests, the government’s approval rating fell to an all-time low of 12% (86% disapproval) (AP, 2017a). López Obrador leveraged continued criticism of the government’s handling of the protests in the 2017–18 presidential election, although its role in aggravating backlash is unclear.

### Comparison of cases

Policy backlash involved different combinations of conditions and mediators across the cases (Table 3). In Canada, policy action triggered especially negative reactions from politicians and people in provinces aligned with extractive industries within a setting already politicized over fossil fuels. Policy backlash was largely culturally driven through hostile campaigns emphasizing cultural incongruence, leading to changes in provincial leadership through elections, which then fostered further negative campaigns. In France, policy action triggered a massive spontaneous eruption of discontent from an economically precarious and predominantly non-urban population who saw themselves as overlooked. Policy backlash was largely driven by economic and practical conditions, involving direct effects on people and amplified in a setting of eroded political trust and a tradition of confrontational protest. In Mexico, policy action triggered widespread pushback from poor and economically insecure people in a context with chronic poverty and inequality. Policy backlash was largely economically and culturally driven, involving not only direct and differential impacts but also ideological divisions over economic liberalization within a setting of fossil fuel hegemony.

**Table 3:** Comparison of the three illustrative cases

Case	Forms of incongruence	Focus of conditions	Role of mediators	Character of backlash
1. Canada	Economic Cultural Practical	Direct impacts on target groups: *Perception of excessive costs* Clash with political beliefs* Differential impacts for target groups: *Perception of unfairness* Threat to group identity* *Unfeasible/impossible expectations* *Discomfort with new practices*	*Media discourse* and political opportunities* amplify incongruence	Policy action reverberates within a setting already politicized over fossil fuel production
2. France	Economic Cultural Practical	Direct impacts on target groups: Perception of excessive costs* Differential impacts for target groups: Perception of unfairness* *Threat to group identity* Unfeasible/impossible expectations* Wider impacts on society: *Misfit with broader cultural values*	Political trust* and *media discourse* allowed discontent to coalesce	Policy action triggers spontaneous eruption in a setting of slow-building pressure
3. Mexico	Economic Cultural Practical	Direct impacts on target groups: Perception of excessive costs* Clash with political beliefs* Differential impacts for target groups: *Perception of unfairness* *Unfeasible/impossible expectations* Wider impacts on society: Misfit with economic circumstances* Misfit with broader cultural values*	Political trust* and *media discourse* allowed rapid spread of discontent	Policy action triggers pushback in a setting of chronic inequality and ideological division

*Conditions and mediators that appear to be strongly associated with policy backlash in each case

Policy outcomes varied across the cases, for reasons which themselves varied. In Canada, persistent party polarization (with policy proponents remaining long-committed to the policy) interacted with institutional and legal circumstances (especially the supreme court victory, and heterogeneous support/opposition across provinces) to stabilize the policy despite backlash.^19^ Yet, in France and Mexico, the intensity of backlash led relatively quickly to policy moderation. This difference could be partly because the broad-based character of backlash in France and Mexico created a broad political threat, whereas in Canada backlash was more aligned with party politics and political threat was counterbalanced by potential political benefit. Hence, both the intensity of backlash and its interaction with wider political and institutional circumstances is likely to be relevant in explaining policy outcomes.

Backlash arose in specific and embedded ways within the three cases, highlighting the need for configurational explanations. This resonates with observations from both policy conflict theory (Kagan et al., 2023; Weible & Heikkila, 2017) and social movement theory (McAdam et al., 2001) that contention is often complex and multi-causal. Our approach thereby informs further comparative empirical analysis and theorizing, such as the possible development of mechanisms (i.e., transferable causal chains) explaining policy backlash. For example, the case of Canada could suggest a cultural-politicization mechanism, the case of France a pressure-eruption mechanism, and the case of Mexico a deprivation-outrage mechanism. Yet, further case-specific work would be needed to develop and test such mechanism-based explanations.

While our analysis has focused on cases of backlash, it is also helpful to consider cases of non-backlash in comparison. One possibility is to consider why backlash has not occurred in other places despite the plausible potential for it. For example, Germany’s coal exit law provides a relevant case. In 2019 a multi-actor Coal Commission^20^ recommended coal phase out, and legislated was passed in 2020 to close coal-fired power plants and end coal production by 2038, with compensation and adjustment for affected regions and workers (Hauenstein et al., 2023). Despite not involving carbon pricing, it is a case of hard policy in a context where backlash could be expected due to capacity and incentives to mobilize. In recent years there has been backlash on other issues including a proposed heat pump law (von der Burchard, 2023), combustion vehicle phase out (Posaner & Cokelaere, 2023), and agricultural environmental policy (Moulson, 2024). Even though the coal exit law remains contested (Raitbaur, 2021), and prior coal closures have impacted local voting preferences (Stutzmann, 2025), it has so far not experienced major public backlash. The policy making approach involving stakeholder representation and policy design involving compensation and structural adjustment (Bang et al., 2022) may have helped to reduce potential incongruence (e.g., concerning *perceptions of unfairness* and *threat to group identity* in certain regions, and even *misfit with broader economic circumstances* considering pressure from the energy crisis linked to Ukraine War).

Another possibility is to consider why backlash has not occurred at other times in the same place. For example, France’s earlier experiences with carbon taxes (prior to the YVM) raise the question of why backlash happened when it did. Attempts to introduce a carbon tax in 2000 and 2009 were withdrawn following some contestation but did not spark a mass movement or other extraordinary reactions (Rocamora, 2017). The carbon component that later triggered the YVM was in place for four years before the eruption of backlash but had been largely invisible due to its integration into existing taxes, incremental design, and low oil prices (Durand, 2018; Rocamora, 2017). Thus, the *perception of excessive costs* and the *perception of unfairness* were not present in the same way as with the YVM (Durand, 2018). Perhaps also lacking earlier was a crystallized *threat to group identity,* as sparked through the petition in the YVM case which coalesced discontent helping to create a shared sense of adversity.

Ideally, comparing the presence/absence of backlash requires sampling from a consistent population where backlash could be plausibly expected. For example, Anisimova and Patterson (2024) systematically sample cases of hard climate policies in OECD countries to examine the prevalence of backlash. Other possibilities could be to compare all countries that have introduced carbon taxes/pricing^21^ and track the policies over time to identify if and when backlash occurred, or compare all countries that have introduced fossil fuel phase outs, for example. Our approach as illustrated here could be employed for doing so.

## Conclusions

Policy backlash needs to be brought into policy sciences to enable comparative empirical analysis. We contribute to this task for the specific domain of climate change policy. By articulating potential socio-political conditions under which policy backlash occurs, our approach helps to understand contextually embedded eruptions of backlash. This could also enable configurational analysis of combinations of conditions driving backlash, and thereby, mechanisms of backlash to climate policy. While our focus is on climate policy (specifically, carbon pricing/taxation), our approach could potentially be adapted to other policy types and domains by considering similar dimensions of incongruence in domain-specific ways.

Our illustrative analysis suggests a need for attention to diverse ways in which incongruence might arise, involving factors both proximate and remote to the policy process. This includes sub-threshold pressures built up over long periods (e.g. economic inequality, cultural disaffection), which may not be readily visible but could become consequential at a certain moment (Pierson, 2003), although mechanisms would still need to be posited for how this occurs (McAdam et al., 2001). It thereby suggests a need to expand policy-centered analysis towards a policy-in-context perspective, broadening the scope of analysis to interrogate how policy action can trigger backlash for diverse and multi-faceted reasons. While this does not make analysis easier, it offers tools for understanding when and why negative reactions to policy action occur.

For climate policy specifically, our approach suggests a need for attention to a diverse range of factors in policy design, enactment, and post-enactment to deal with the challenging politics of hard climate policy. This goes beyond the now generally accepted need to address cost distributions and fairness, to also consider cultural and practical dimensions, and how these produce reactions to policy action among target groups and wider audiences. This is also important considering the contextual embeddedness of policy action, even when it may be hard to know whether to expect backlash. Climate policy debates have been shifting from an emphasis on pricing/taxation to investment (e.g., United States’ Inflation Reduction Act) and structural adjustment (e.g., European Green Deal), in no small part due to fears of backlash. However, carrots may not always be fiscally possible, such policy may not be immune from backlash, and there may be many ways in which hard policy remains crucial to realizing societal transitions. Thus, rather than avoiding hard policies,^22^ it would be fruitful to consider why they provoke negative reactions in specific situations, and whether and how potentially diverse forms of underlying discontent could be addressed.

Our approach could also help to inform strategic thinking on policy action to anticipate or avoid backlash, or at least to gain insight into its likelihood. Policy backlash will arguably remain difficult to anticipate, yet it is important to consider during policymaking. For example, Patashnik (2023, p.182–5) suggests several possible strategies including designing policies to make it more difficult for organized interests to oppose, attending to those who bear losses, balancing concerns of different supporters, and embedding a policy over time even if it appears slow at the outset. We would add that attention to narratives and meanings of policy action and the complex ways that it intersects with peoples’ everyday lives are also important. This could offer innovative areas for further strategies – beyond the immediate scope of policy design yet within broader struggles over policy enactment and post-enactment as it unfolds in society. Our approach can therefore enable broad insight on the “room to maneuver” in policy processes (Karapin, 2018), for example, supporting horizon scanning to identify where conditions of incongruence could arise. Importantly though, policy backlash is likely to require relatively extreme conditions of incongruence. This may be unusual, but it is also why, in such cases, any early signals and guidance on proactive strategies would be helpful. At the same time, this poses an intriguing and pressing research agenda on understanding and responding to policy backlash in a time of increasingly contentious and intractable contemporary policy challenges.

## Footnotes

1 Coercive action is a basic capacity of government including for addressing collective problems such as climate change (Mansbridge, 2012, p.4). We focus on hard/coercive policy rather than incentive- or voluntarism-based policy but note that backlash could occur for these latter types especially if seen as coercive/unjust (e.g., affecting resources or status).

2 Although notably Jones et al., (2019) consider conservative counterreactions to policy expansion combining punctuated equilibrium theory and policy feedback.

3 Whereas policy conflict can occur across all policy process stages (agenda setting, design, adoption, post-adoption).

4 Also following Weible and Heikkila (2017, p.29–30).

5 Patashnik (2023) uses the term ‘countermobilization’ which largely overlaps but could imply coalitional responses. ‘Reactive mobilization’ allows for open, spontaneous reactions where actors – while united in seeking to reverse a policy – may not share alternative policy preferences.

6 Ragusa and Birkhead (2020, p.7) define repeal as “an effort to ‘nullify’, ‘annul’ or ‘undo’ a previously enacted law”.

7 Which could range from repeal, weakening, or delay, to no change, or even policy consolidation (Patashnik, 2023).

8 For example, partisanship (Ragusa & Birkhead, 2020) and “changes in the partisan composition of congresses” (Berry et al., 2010) play a key role in legislative repeals in the United States, suggesting a coalitional explanation. It would be intriguing to consider which cases also involve backlash.

9 For example, Patashnik (2023, p.14) notes that elites may “stoke, amplify or suppress public backlashes”, and Mildenberger (2020, p.4) notes that “policy losers [may] mobilize conflict into the public domain”.

10 Scheduled to reach CA$170 per tonne of CO2e by 2030 (ECCC, 2023).

11 For example, outright exemptions for agricultural fuels (Rack, 2022), its patchwork approach functionally inviting provinces to adopt the least demanding option for them (Harrison, 2019), and this flexibility leading to inconsistent exemptions/compliance for emissions-intensive industry (Rack, 2022; Sawyer et al., 2021).

12 For example, following a rally in Regina in 2019, conservative Premier of Saskatchewan Scott Moe not only used the standard line about the “job-killing” carbon tax, but also added that he “…wasn’t stumping for the federal Conservatives, but the people of Saskatchewan” (Baxter, 2019).

13 Our translation.

14 Including being adjusted annually for inflation (Arlinghaus & van Dender, 2017).

15 For instance, large dolls were hung on some bridges and streets stating: “The CFE [i.e., Federal Electricity Commission] also robbed me” (Ríos, 2017).

16 The Centre-left Party of the Democratic Revolution (PRD) labelled the price a national emergency, while the conservative-right National Action Party (PAN) requested that the government (PRI) “find alternatives to alleviate the impact that the increases will have on Mexican families.” (AP, 2017b).

17 Our translation.

18 Our translation.

19 Although, as noted in Sect. "Canada: Greenhouse Gas Pollution Pricing Act", the consumer carbon price component was eventually neutralised in 2025.

20 German Commission on Growth, Structural Change and Employment.

21 There are approximately 40 countries with emissions trading systems or carbon prices implemented, as well as in subnational jurisdictions within these and other countries, and many more under consideration (World Bank, 2024).

22 After all, “Making good policy decisions may require taking politically perilous actions, such as imposing losses on powerful constituencies” (Patashnik, 2023, p.16).
